# Implementation of integrated care for type 2 diabetes Mellitus and Periodontitis in Germany: study protocol for a practice-based and cluster-randomized trial

**DOI:** 10.1186/s12903-024-04672-1

**Published:** 2024-08-02

**Authors:** Patrick Hennrich, Annika Queder, Attila Altiner, Sinclair Awounvo, Jan Dyczmons, Julian Eigendorf, Stella Erdmann, Thomas Grobe, Andreas Gutscher, Nicole Herzig, Søren Jepsen, Nadja Kairies-Schwarz, Olivier Kalmus, Fabian Kliemannel, Sara Santos, Patrizio Vanella, Michel Wensing, Stefan Wilm, Stefan Listl

**Affiliations:** 1 Heidelberg Institute of Global Health Section for Oral Health, Heidelberg University Hospital, Im Neuenheimer Feld 130.3, 69120 Heidelberg, Germany; 2 Department for General Practice and Health Services Research, Heidelberg University Hospital, Im Neuenheimer Feld 130.3, 69120 Heidelberg, Germany; 3 Düsseldorf University Hospital Institute for Health Services Research and Health Economics, Moorenstr. 5, 40225 Düsseldorf, Germany; 4 aQua-Institut für angewandte Qualitätsförderung und Forschung im Gesundheitswesen GmbH, Maschmühlenweg 8-10, 37073 Göttingen, Germany; 5 Heidelberg University Hospital Institute of Medical Biometry, Im Neuenheimer Feld 130.3, 69120 Heidelberg, Germany; 6 Techniker Krankenkasse, Bramfelder Str. 140, 22305 Hamburg, Germany; 7 Department of Periodontology, Operative and Preventive Dentistry, Bonn University Hospital, Welschnonnenstr. 17, 53111 Bonn, Germany; 8 Düsseldorf University Hospital Institute of General Practice, Moorenstr. 5, 40225 Düsseldorf, Germany

**Keywords:** Screening, Periodontitis, Diabetes, Integration, Implementation, Germany, General practice, Dental practice, Oral health

## Abstract

**Background:**

Type 2 Diabetes mellitus (T2DM) and periodontitis share common risk factors and influence one another. However, primary care and oral health care continue to operate separate from each other and fail to synchronize care for patients with T2DM and periodontitis. The purpose of this practice-based trial is to evaluate the implementation of a new integrated care pathway for patients with T2DM and periodontitis. The new approach integrates a screening for T2DM risk in dental care settings in patients with periodontitis, a screening for periodontitis risk in primary care settings in patients with T2DM, and mutual referrals between dentists and primary care physicians.

**Methods:**

Two practice-based studies will be carried out in parallel: (i) In dental care settings: a practice-based, multi-centric, cluster-randomized, controlled trial with a control and an intervention group; (ii) in primary care settings: a practice-based, multi-centric, non-randomized, controlled trial with a synthetic control group calculated from claims data. Following a two-step recruitment approach, 166 dentists and 248 general practitioners will be recruited, who themselves will recruit a total of 3808 patients in their practices. Patient data will be collected at baseline, 12 months, and 24 months after study enrollment. The evaluation comprises: (i) impact evaluation, using a hierarchical linear mixed model; (ii) process evaluation, based on surveys alongside the trials; (iii) economic evaluation. In addition, a Discrete-Choice-Experiment will identify provider’s payment preferences for the new care approach.

**Discussion:**

Upon successful implementation, the intervention will enable health care providers to detect a risk for T2DM and periodontitis in patients at an early stage, thus providing patients an opportunity for timely diagnosis and therapy. Ultimately, this can lead to increased quality of life and reduced health care expenditures. On a methodologic level, the project provides novel insights into a complex intervention on the intersection of general practice and dental care.

**Trial registration:**

The study was prospectively registered at the German Clinical Trials Register (https://drks.de/search/de/trial/DRKS00030587) on 3. July 2023 under ID “DRKS00030587“.

**Supplementary Information:**

The online version contains supplementary material available at 10.1186/s12903-024-04672-1.

**Table:** 

Contributions to the literature	
- First large-scale practice-based trial on the implementation of integrated care for type 2 diabetes mellitus and periodontitis in both the primary medical care and the dental care settings. - Novel methodological approach to evaluate implementation of integrated care pathways in previously asynchronous care settings (dentistry and primary care). - Unique comparison of similarities and differences between physicians and dentists with respect to barriers and facilitators for implementation of interprofessional care integration. - Identification of provider’s payment preferences as integral component for implementation. - Unique insights into feasibility of implementation research in dental practice settings.	

## Background

The 2021 WHO Resolution on Oral Health resolution emphasizes the need for better integration of oral health and primary health care [1].

Previous evidence suggests that oral diseases and other noncommunicable diseases occur in parallel [2, 3]. Periodontitis is 2–3 times more prevalent in patients suffering from type-2 diabetes mellitus (T2DM) [4]. T2DM-patients have a higher periodontitis risk [5–7]. Among T2DM patients, periodontitis therapy was shown to reduce HbA1c by 0.6%-points up to 12 months [8] as well as healthcare costs [9, 10]. On the other hand, glycemic control in periodontitis patients may be improved by periodontitis therapy [7, 11, 12].

This shows that better integration of T2DM and periodontitis care offers potential to reduce disease burden and costs - yet, the diagnosis and treatment of T2DM and periodontitis are still disintegrated in Germany, which is the context for this study [13]. The number of undiagnosed T2DM-cases amounts to around 2 million and it takes an average 8 years until T2DM is diagnosed [14]. There is also a discrepancy between need and utilization of periodontitis care: While 10 million people suffer from severe periodontitis, there are 1 million reimbursed treatment cases per year [15]. These gaps between need and utilization give raise to avoidable disease burden and costs. To this end, the present paper describes the protocol for practice-based studies on integrated T2DM and periodontitis care in Germany.

### Description of the implementation approach for integrated care

The **Dig**itally **In**tegrated T**2**DM and **Perio**dontitis (DigIn2Perio) study aims to evaluate new interprofessional care pathways as an implementation strategy to achieve integrated care for T2DM and periodontitis. The care pathways include (different from usual care):

- T2DM screening among periodontitis patients, using the FINDRISK-questionnaire [16, 17].
- Periodontitis screening among T2DM patients, using the periodontitis risk score of the German Society for Periodontology [18].
- Tailored patient information about the interplay between T2DM and periodontitis.
- Mutual referrals between primary care physicians and dental practitioners.
- Digitally supported data capture and exchange via the German Telematics Infrastructure [19].

**Fig. 1:**
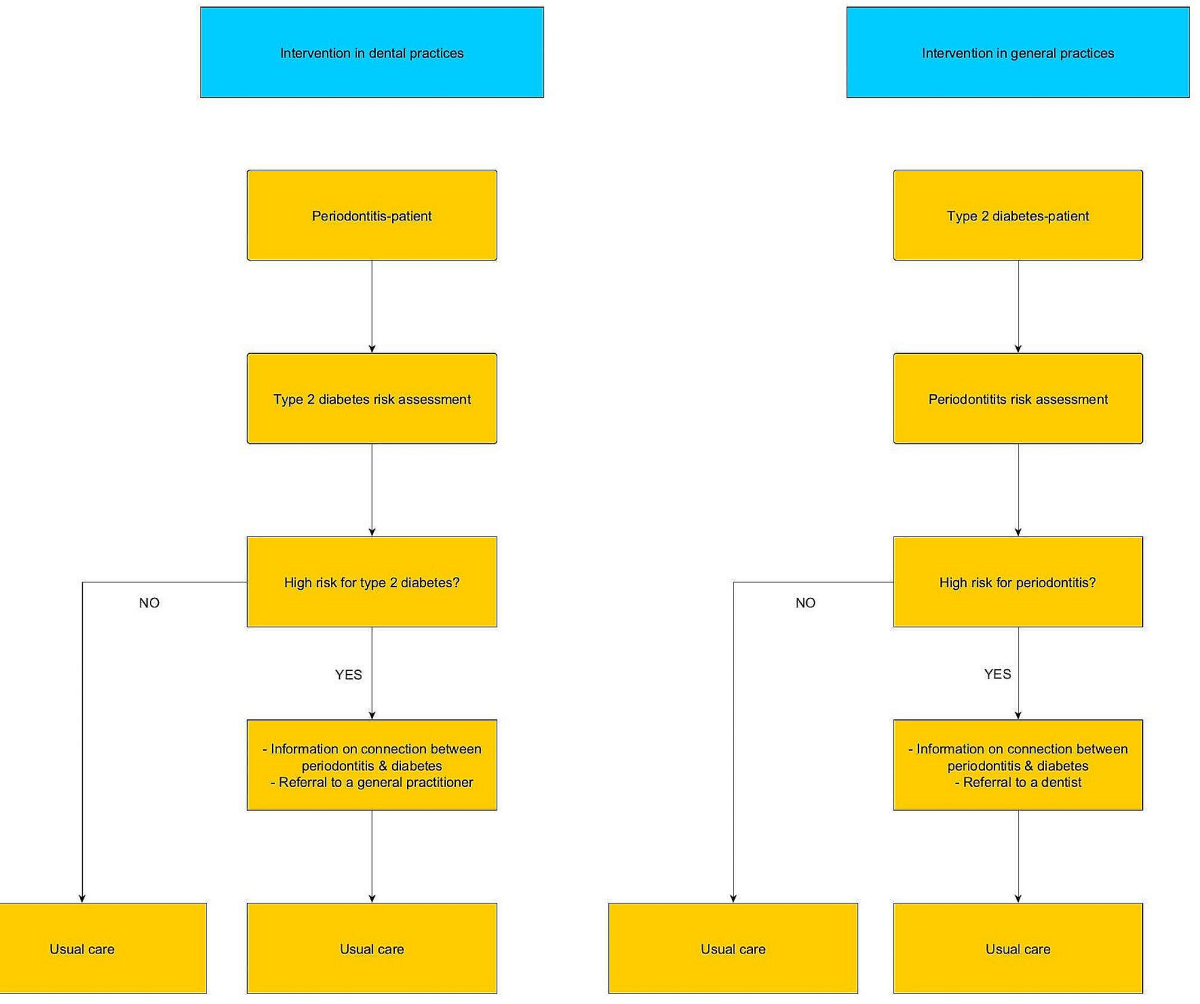
Intervention concept: (**a**) initiation in dental practice; (**b**) initiation in primary care practice

Figure 1 gives an overview of the implementation approach for integrated care:

### Research questions

#### Primary question

Does the new approach for implementation of integrated T2DM and periodontitis care result in:

(i) lowered HbA1c among T2DM patients;
(ii) additional T2DM diagnoses among periodontitis patients?

#### Secondary questions

Does the new approach for implementation of integrated T2DM and periodontitis care:

- lead to better periodontal health in response to dentist-initiated T2DM care?
- improve patients’ quality of life?
- lead to more risk-tailored utilization of care and medication?
- lend itself for practice implementation as anticipated? (process evaluation)
- provide good value-for-money?
- evoke specific provider payment preferences?

## Materials & methods

### Endpoints

#### Primary endpoints

##### In general practitioner (GP) practices

HbA1c reduction among patients in the T2DM Disease Management Program (T2DM-DMP) who had not received periodontitis therapy within the past 12 months. Recorded by medical assistants at: T0 (recruitment); T1 (after 12 months); T2 (after max. 24 months). [primary data]

##### In dental practices

New T2DM diagnoses among periodontitis patients who had not been diagnosed with a diabetes condition before. Recorded by dental assistants at T1 (after 12 months). [primary data]

#### Secondary endpoints

##### In GP practices

- Utilization of periodontitis therapy (yes/no) [primary & claims data].
- T2DM medication (no medication, oral medication, insulin injections) [primary & claims data].
- Patients’ quality of life [primary data].

##### In dental practices

- % of teeth with pocket probing depth > 4 mm and bleeding on probing (BOP) [primary data].
- T2DM medication (no medication, oral medication, insulin injections) [primary data].
- Patients’ quality of life [primary data].
- Utilization of T2DM care (blood sugar-parameters, inclusion in T2DM-DMP) [primary data].
- Utilization of periodontal care [claims data].

## Evaluation approach

### Impact evaluation

#### Study in general practices and dental practices

The GP study is a practice-based, multi-center, non-randomized, controlled trial. The intervention group will be recruited in general practices, the control group will be calculated from insurer claims data as well as from DMP documentation data across insurers (triangulation in comparison to the insurer-data) parallel to the clinical intervention group.

The dentist study is a practice-based, multi-center, cluster-randomized, controlled trial with a control and an intervention group. Both groups are recruited in dental practices. Patients are clustered in practices, hence a randomization on cluster-level was deemed appropriate.

Both studies will be carried out in the German states of Baden-Württemberg and North Rhine-Westphalia.

##### Inclusion & exclusion criteria

Table 1 illustrates inclusion & exclusion criteria for practice personnel and patients alike in the main study.

**Table 1:** Inclusion and exclusion criteria of the main study

	Practice & practice personnel	Patients
**Inclusion criteria**	- Legal age - Ability to consent	
	- Established, licensed physicians and assistants - Based in Baden-Wuerttemberg or North Rhine-Westphalia - Working computer system with internet connection - Patients physically visit the practice	- Insured with a statutory health insurer - GP-study: In T2DM-DMP in 4 of 5 past quarters - Dentist study: Need for systemic periodontitis therapy
**Exclusion criteria**	Persons who : - Live in a nursing home - Have a legal guardian - Are not of legal age - Are cognitively or linguistically incapable to participate	
	- Are not licensed as physician - Don’t practice in Baden-Württemberg or North Rhine-Westphalia	- Are not insured with a German statutory health insurer (or not entitled to care that exceeds basic health care coverage) - GP-study: a periodontitis therapy in the past 12 months - Not in the T2DM-DMP in 4 of 5 past quarters - Dentist study: Previously diagnosed with diabetes

##### Randomization procedure

###### General practitioner study

To ensure comparability of the (calculated) control group and the (recruited) intervention group, triangulation will be performed using insurer claims data and DMP-documentation data across insurers (pseudonymized data on person-level). Consent will be obtained from the Federal Office for Social Security in Germany to acquire and use the data without needing informed consent from each patient.

Contrary to insurer claims data, DMP data lacks information on secondary endpoints. However, representativeness of DMP data that has been gathered across all insurers is higher than that of data from a single insurer. Triangulation can improve validity of control group data and its comparability to the intervention group (in the sense of an emulated counterfactual with demographic characteristics of the intervention group). For intervention patients, there will be survey data equal to the claims data as well as additional survey data.

###### Dentist study

Dental practices are assigned to a control and intervention group in a 1:1 ratio using a central, web-based tool (randomizer.at) to avoid any manipulation and ensure concealed allocation. Randomization will be stratified by state and use a block-wise randomization process with a varying length of blocks to arrive at groups of equal size and avoid selection bias. Randomization will take place on the practice (cluster) level, so all participants within a given practice will either be part of the intervention or the control group. The randomizing tool will be administered by the evaluating Institute of Medical Biometry at the Heidelberg University Hospital.

##### Sample size calculation

###### General practitioner study

Sample size calculation refers to the primary endpoint and is based on a t-test (α = 0.05; power = 0.8). We assume that HbA1c in the intervention group will decrease on average by 0.2%-points (standard deviation (sd) of 2.3) compared to the control group. The estimator results from the assumption that at least 50% of T2DM-patients have a need for periodontitis therapy [15, 20] and that they’re able to lower their HbA1c by 0.6%-points through therapy [8]. Out of these patients, an assumed 66% will use the referral to a dentist [21]. To account for cluster effects (patients clustered in practices), an intra-class-correlation of 0.03 and a cluster size of 10 patients per practice is assumed. For the control group calculated from claims data, we assume that it contains at least double the number of patients than the intervention group (allocation in a 2:1 ratio for control vs. intervention group). This results in a sample size of 1980 intervention patients (198 practices, 10 patients each) and 3960 control patients. With an assumed drop-out rate of 20%, there will be 2480 intervention patients (248 practices, 10 patients each) and 4950 control patients. Sample size calculation was performed using PASS v16.0.3.

###### Dentist study

Sample size calculation refers to the primary endpoint and is therefore based on a Z-Test with non-pooled variance. We assume that, compared to the control group, the rate of new diabetes diagnoses will be 0.1 in the intervention group during the next 4 quarters [22]. For the control group we assume a rate of 0.05 (calculated from an internal analysis of insurer-data for the areas the study will be performed in). It is therefore assumed that the intervention allows for an additional 5% of diabetes diagnoses. Sample size is calculated with α = 5% and power = 80%. Patients are clustered in practices, so an intra-class-correlation of 0.03 and a cluster size of 8 patients per practice is assumed. Assuming an allocation ratio of 1:1 for control vs. intervention group, this results in a sample size of 528 patients (66 practices, 8 patients each) per group. With an assumed drop-out rate of 20%, sample size in each group will be 664 patients (83 practices, 8 patients each), adding up to a total of 1328 patients in 166 practices. Sample size calculation was performed using PASS v16.0.3.

##### Recruitment

Recruitment in the GP study will be actively supported by the Association of Statutory Health Insurance Physicians in the state of Baden-Württemberg (KVBW), the research network of general practitioners in the state of North Rhine-Westphalia (HAFO.NRW), the network of research practices at the Heidelberg University Hospital (FoPra.HD) and the Association of General Practitioners in Baden-Württemberg. Recruitment in the dentist study will be supported by the Association of Statutory Health Insurance Dentists in the state of Baden-Württemberg (KZVBW) and North Rhine-Westphalia (KZVNR).

We will distribute a short invitation letter or fax containing an overview of the study (contents, duration, conditions, compensation for expenses). Practice owners will be asked to indicate whether they’re interested in participating and return the letter via fax or e-mail. Phone calls will be arranged to provide further information to practices that are interested in participation. Information material will be provided to the practice beforehand. The practice receives:

- Detailed study information.
- Informed consent forms for the practice owner, the assistant who will take care of the project and for possible further physicians in the practice.
- The required authorization form to include the practice in the care contract with the insurer (intervention groups only).

As soon as the authorization form and each form of consent is returned dated and signed by the respective person, the practice is included in the study. They then receive:

- Their unique alphanumeric practice ID.
- A study folder containing countersigned copies of the practice documents as well as guidance and other legally required documents.
- A patient folder containing the documents to perform the study in the practice (background information for patients, informed consent forms, questionnaires).
- Log-in data for the research database where practice and questionnaire data will be documented.

When first using the research database, practices need to enter basic data on the practice (size, type, population density, year of establishment, patients per quarter). Afterwards, they may start recruiting patients.

Recruitment and data collection/transfer will be performed by the medical/dental assistant named as the responsible person for the study within the practice. They receive an online video tutorial on how to implement the components of the study.

Medical/dental assistants identify eligible patients according to the inclusion criteria (Table 1) when they visit the practice. They verbally inform these patients about the study, hand out the documents necessary for participation and respond to potential questions. Patients partake in the study by signing the informed consent form. The form will be countersigned by the assistant and the patient receives a copy. Directly afterwards, the baseline survey (T0) takes place.

##### Digital patient record

In both parts of the main study, before T0, the medical/dental assistant will ask the patients whether or not they use a digital patient record or if they wish to activate this feature. If they use one and the participant agrees, diagnoses and risk scores will be deposited in the digital patient record in addition to the regular patient record. If patients wish to activate their digital record, T0 will take place as planned and the respective data will be deposited in the digital record retrospectively after activation. If patients do not wish to use the digital record or it cannot be used for technical reasons, data will be stored in the regular patient record only.

##### Baseline survey (T0)

Patients in both studies receive a written questionnaire on sociodemographic data and a self-disclosure of medical conditions. The questionnaire further contains a question on current, subjective health, translated from the publicly available, validated Short Form 36 (SF-36)-questionnaire by RAND Corporation [23]. Additionally, the questionnaire contains the respective questions for periodontitis/T2DM risk assessment (in the dentist study, this is only the case for the intervention group), so patients may calculate their risk score. The medical/dental assistant will be available for questions during this process. The further course differs depending on the study and risk-score.

###### T0 in general practices

**Fig. 2:**
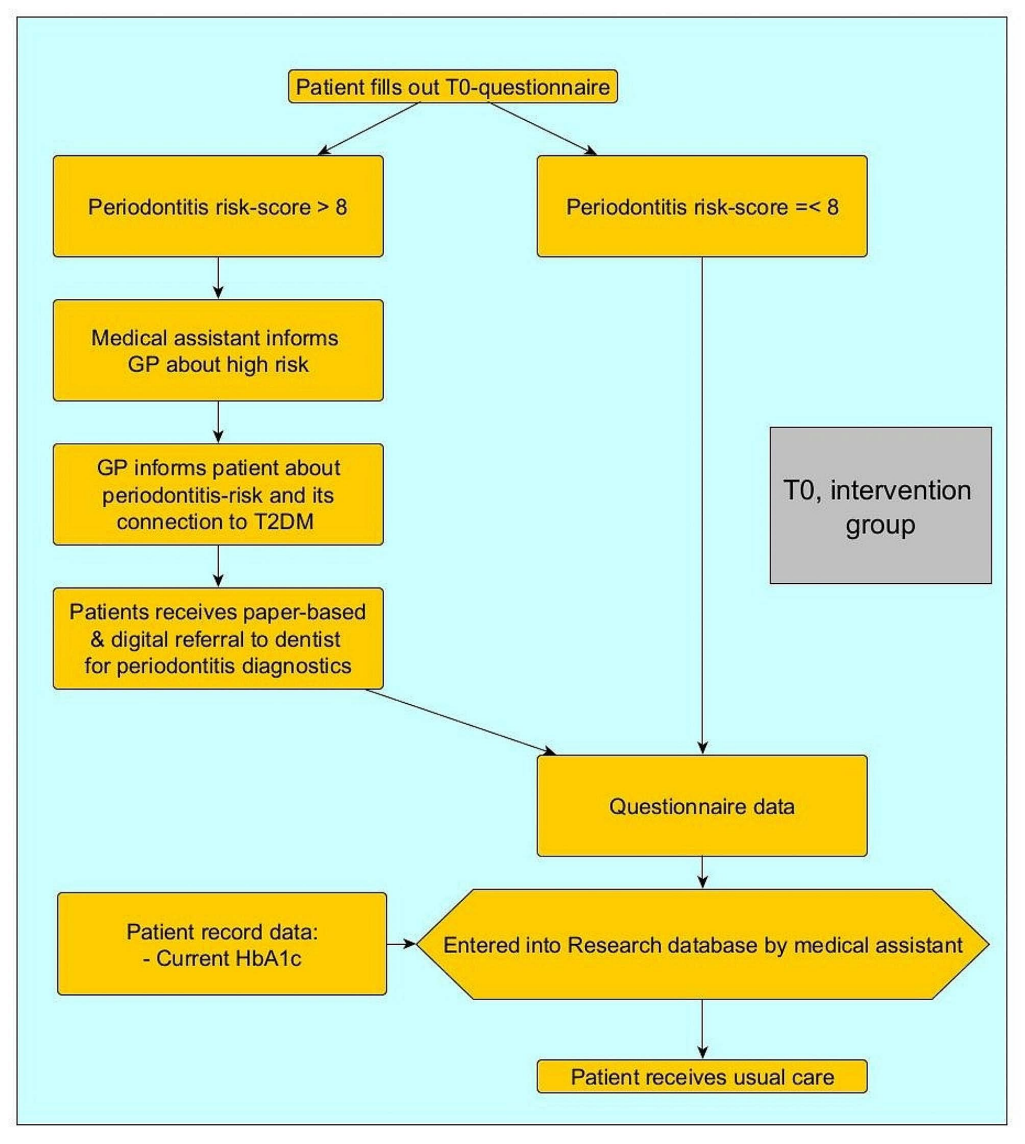
Baseline survey T0 in general practices

Figure 2 provides an overview over T0 in general practices after patients receive the questionnaire described above.

After T0, all patients receive usual care. During the following, regular DMP appointments, the GP will approach high-risk patients regarding the dentist appointment and potential measures that the dentist took. The follow-up survey T1 will take place 12 months after recruitment.

###### T0 in dental practices

**Fig. 3:**
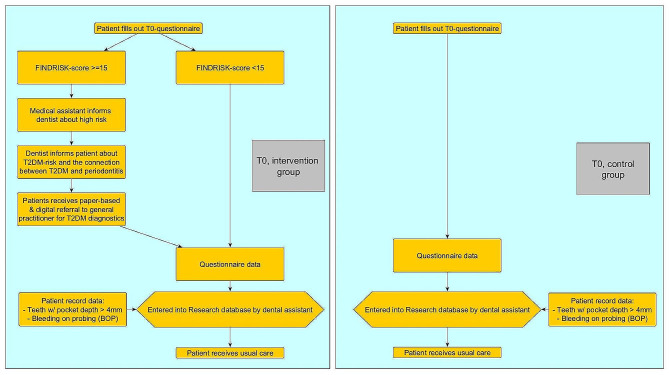
Baseline survey T0 in dental practices

Similar to the general practice study, the study pathways differ depending on the risk score of each patient. Additionally, the pathways differ between intervention and control group, as it can be seen in Fig. 3:

After T0, all patients receive usual care. During the following, regular dentist appointments, the dentist will approach high-risk patients in the intervention group regarding the GP appointment and potential measures that the GP took. The follow-up survey T1 will take place 12 months after recruitment.

##### Survey T1 (first follow-up)

The medical assistant in the general practice identifies all participating patients during a regular DMP-appointment. In the dental practice, the dental assistant identifies all participating patients during a regular appointment. Patients are asked to fill out another written questionnaire on their health and a potential periodontitis/T2DM diagnosis and therapy. Subjective health is once again assessed using the first question of the SF-36. To allow for a comparison with the patient’s subjective health at T0, question 2 of the SF-36 will be asked as well.

In the GP-study, the GP asks the patients about their potential periodontitis diagnosis and/or therapy. The primary endpoint measured during the regular DMP-appointment as well as the questionnaire data (including secondary endpoints) for T1 will be transferred to the research database by the medical assistant.

In the dentist study, the dentist in both control and intervention group asks the patients about their potential T2DM diagnosis and/or therapy. The current periodontal status and the periodontal services provided will, together with the questionnaire data, be transferred to the research database by the dental assistant.

The second, and final, follow-up survey takes place 6 to 12 months after T1, depending on when the patient was recruited for T0.

##### Survey T2 (second follow-up)

The medical/dental assistants identify patients similar to T1. Patients in general practices are asked to fill out another written questionnaire on their health and a potential periodontitis diagnosis and therapy as well as the first two questions of the SF-36. These data and the current HbA1c-parameter will be transferred to the research database. T2 serves the purpose of checking on a permanent reduction of the HbA1c-parameter and to get a long-term picture of the secondary endpoints as well.

Patients in the dental practice are asked to fill out another written questionnaire on their health and a potential T2DM diagnosis and therapy as well as the first two questions of the SF-36. These data and the current periodontal status and the periodontal services provided will be transferred to the research database. T2 here serves the purpose of getting a long-term picture of the secondary endpoints.

##### Financial incentives and reimbursement for the provision of care

All participating practices receive a compensation of 250 Euros for participation. The medical/dental assistant responsible for the execution of the study within the practice receives 25 Euros for each patient recruited. Additionally, the practice receives 180 Euros for each recruited patient who is fully documented (survey and data transfer have taken place at T0, T1 and T2). Intervention patients receive the benefit of potentially learning about a present periodontitis/T2DM condition earlier than in usual care. They receive no monetary incentives.

Services in the intervention groups exceeding usual care (risk assessment, informing patients about their risk, doctor’s consultation, referral to the dentist/GP) are covered by the study budget and will be reimbursed with 30 Euros per patient. Practices are prohibited from billing these costs with the patient or health insurer.

### Process evaluation

The process evaluation is based on a written survey at two points in time and semi-structured interviews with care providers from both studies.

#### Written survey

All participating care providers in the intervention groups receive a written, pseudonymized questionnaire at the beginning and end of the observation period. Data will be stored and analyzed separately from the main study. The process evaluation is expected to cover 662 care providers from 331 intervention practices. With an assumed response rate of 60% there will be 397 completed questionnaires for each of the two measurement points. Topics will be acceptance of the implementation strategies (tutorials, financial aspects and billing, organizational support) as well as the implementation fidelity. Perceived barriers and enabling factors will be determined through a suitable framework, such as the CFIR-framework [24] or the TICD-framework [25] in a pre-post-comparison.

#### Interviews

A targeted sample of 40 care providers (equally divided into dental and general practices) will be invited to an additional phone interview during the intervention period. This sample size is usually sufficient to reach topical saturation and saturation of contents in the qualitative analysis. Interviews are planned to last 30–45 min and will be conducted using a semi-structured interview guideline that covers barriers and enablers of the implementation and criteria for maintaining the intervention as well as time expenditure for the study. Furthermore, factors influencing the motivation to participate in the study from the participants’ views will be explored. For the interviews there will be a separate form of informed consent. Interviews will be audio-recorded, transcribed verbatim and stored on a secure server. Transcripts will be pseudonymized.

### Economic evaluation

The economic evaluation focuses on costs and health benefits of the intervention compared to usual care. It uses trial data as well as claims- and DMP-data. Furthermore, there will be a discrete choice experiment (DCE) at the intersection of economic aspects and knowledge transfer regarding the implementation of new types of care (see Appendix 1 for details).

## Statistical analyses

### Analytic population

Primary statistical analysis is based on the full analysis set (FAS) following the intention-to-treat (ITT) principle and therefore being performed in accordance with current guidelines [26]. FAS includes all recruited patients (GP study) or those who were randomly assigned (dentist study) to a group, regardless of whether or not they actually participated in the intervention or dropped out of the study later on. This is the primary population for the evaluation of primary and secondary endpoints and study characteristics. To analyze the sensitivity of results, an analysis of the full dataset will be performed in addition. Drop-out and missing values will be accounted for by the intention-to-treat analyses. Missing values will be imputed.

### Statistical hypotheses and analyses

#### General practitioner study

For the primary outcome, we will test the null hypothesis H_0_^HA^: µ_IG_^T1−T0^ = µ_KG_^T1−T0^ against the alternative hypothesis H_1_^HA^: µ_IG_^T1−T0^ ≠ µ_KG_^T1−T0^ on a two-sided significance-level of 5%. µ_IG_^T1−T0^ and µ_KG_^T1−T0^ represent the reduction of the HbA1c-parameter between baseline (T0) and after 4 quarters (T1) in both groups.

Evaluation of the intervention contains descriptive and explorative statistical analyses of gathered study data as well as available claims data. The goal is an independent, scientifically valid and differentiated statement on effectiveness of the intervention regarding a reduction of the HbA1c parameter using an intervention study with parallel control group. The control group will be calculated from insurer claims data as well as DMP-documentation data across insurers. Using their registration number, intervention practices will be removed from the control group data. Study data will be described using descriptive methods.

Primary analysis will be a hierarchical, linear mixed model representing the multi-level structure of the data (level 1: practice, level 2: patient). For the test of H_0_^HA^, the dependent variable will be “reduction of HbA1c between T0 and T1”. The model contains the practice as a random effect and HbA1c at baseline (T0), age, sex, insurance status, state and residential area as fixed effects on the patient level. Here, only variables that are also included in claims data can be used.

It can be assumed that claims data will be complete and missing values will only occur in the additional study data and the questionnaires. Missing values of the primary outcome will be imputed using multiple imputation [27] while accounting for the baseline-variables age, sex, insurance status and residential area. Assumptions on missing values will be analyzed and an alternative procedure to handle missing data, such as an analysis of the full dataset, will be looked into.

Secondary endpoints will be evaluated descriptively and descriptive p-values will be presented with 95% confidence intervals for the corresponding effects. Where appropriate, secondary endpoints will be explored similar to the primary analysis. For binary endpoints, logistic regression analyses will be performed. For continuous endpoints, linear regression analyses will be performed. For the intervention group, additional to the claims data, there will be the data gathered within the study. To analyze their impact on the reduction of the HbA1c-parameter, these data will be included as fixed effects in a linear mixed model in the sub-group of intervention patients.

#### Dentist study

For the primary outcome, we will test the null hypothesis H_0_^ZA^: π_IG_^T1−T0^ = π_KG_^T1−T0^ against the alternative hypothesis H_1_^ZA^: π_IG_^T1−T0^ ≠ π_KG_^T1−T0^ on a two-sided significance-level of 5%. π_IG_^T1−T0^ and π_KG_^T1−T0^ represent the rate of new diabetes diagnoses between baseline (T0) and after 12 months (T1) in both groups.

Primary analysis will be performed using a generalized, logistic mixed model adequately representing the multi-level structure of data (practice on level 1, patients on level 2). For the test of H_0_^ZA,^ the „rate of new diabetes diagnoses between T0 and T1” will be used as the dependent variable. The model contains the practice as a random effect and the group (intervention vs. control), age, sex, insurance status, state and residential area as fixed effects on the patient level.

It can be assumed that claims data will be complete and missing values will only occur in the additional study data and the questionnaires. Missing values of the primary outcome will be imputed using multiple imputation [27] while accounting for the baseline-variables age, sex, insurance status and residential area. To check for the sensitivity of results, assumptions on missing values will be analyzed and an alternative procedure to handle missing data, such as an analysis of the full dataset, will be looked into. Further sensitivity analyses include sub-group analyses for both states and for insurance status.

Secondary endpoints will be presented descriptively first. Where appropriate, secondary endpoints will be explored similar to the primary analysis. For binary endpoints, logistic regression analyses will be performed. For continuous endpoints, linear regression analyses will be performed. For all estimated effects we will present descriptive p-values and 95% confidence intervals. All analyses are performed using a validated R environment with R version 4.0.0 or higher. The primary analysis is independently counter-programmed in SAS version 9.4 or higher.

#### Process evaluation

Quantitative data will primarily be analyzed descriptively for the GP and the dentist study. Categorial variables will be presented in absolute and relative frequencies. Continuous variables will be presented using mean values with standard deviation or median values using interquartile range, minimum and maximum. Differences between groups will be analyzed using variance analyses. Changes between T0 and T1 will be exploratively tested for influences on implementation adherence using regression analyses. Analyses will be performed using IBM SPSS statistics.

The transcribed, semi-structured phone interviews will be analyzed in inductive and deductive phases. In the deductive phase, interview data will be analyzed using a thematic framework analysis to classify and organize data on the base of key topics, concepts and pre-defined categories. Analysis will be performed using MAXQDA.

Qualitative and quantitative data will be integrated using a framework analysis [28, 29]. Topics emerging from the data will be assigned to the identified areas of the framework (e.g. CFIR [24] or TICD [25]). Results from both parts of the process evaluation will then be combined using an integrative analysis [30].

#### Economic evaluation

A cost-cost-analysis will compare total costs for periodontitis- and diabetes-specific treatments in the intervention group with a representative control group. Simultaneously, a corresponding benefit-benefit-analysis will, in the context of a difference-in-differences analysis, analyze subjective health changes of study participants over the course of the study between intervention and control group.

In case of a positive or inconclusive result of these first analyses on the advantages of the intervention, the incremental cost-effectiveness-relation (ICER) of the intervention will be calculated in comparison to usual care and based on DMP and claims data as well as results from the main and process evaluation. Furthermore, there will be calculations on costs and effects of the transfer of the intervention to usual care. To calculate the ICER, differences in the costs will be compared with differences in health effects. Costs of usual care will be calculated from claims data [10]. Additional costs caused by the intervention will be estimated on the base of documented costs from the study and approximated personnel costs from the results of the process evaluation (time spent for the intervention multiplied with usual rates for personnel costs). All analyses will be performed using a validated R-environment with R version 4.2.2. or higher.

## Termination criteria

Individual termination criteria for practices and patients as well as overall termination criteria for the study are shown in Table 2:

**Table 2:** Termination criteria for DigIn2Perio

Practices	Patients	Study
- Revocation of the consent to participate - Permanent loss of the infrastructure required to perform the study - Permanent closing - Relocation to a state outside of Baden-Württemberg or North Rhine-Westphalia - Transfer to an owner who is not willing to participate or who fulfills at least one exclusion criterion - Permanent loss of responsible assistant if no other person is willing/able to continue	- Revocation of the consent to participate - Death - Occurrence of at least one exclusion criterion in the course of the study - Permanent change of practice	- Permanent loss of the study funding - Loss of and/or actual incapacity to act at the study administration - Loss of project partners whose contributions are indispensable for the study, unless these contributions can be acquired elsewhere - Changes in legal regulations or the security situation that make it impossible to continue

## Expected use and insights of the study

The study will provide the following insights and have the following expected use for improved patient care within the study and healthcare more generally:

- Quality of care: Improved care for T2DM and periodontitis is known to reduce morbidity and increase quality of life in patients. The participatory aspects of the new care approach will contribute to therapeutic success.
- Cost-efficiency: Periodontal care for T2DM patients are expected to yield a noticeable reduction of T2DM-associated care costs [9, 10, 31, 32]. Care costs per diabetes patient have been shown to be reduced by about 600 Euros within three years in response to periodontitis care [10].
- Elimination of care deficits: T2DM/periodontitis conditions that are currently diagnosed late or not at all will be detected earlier due to the targeted screening measures. Furthermore, we expect the intervention to lead to an ongoing improvement in oral health behavior as well as aftercare.
- Due to operationalization of structured and data-supported interdisciplinary care pathways, we expect DigIn2Perio to provide a valuable contribution to enhancing interprofessional and intersectoral collaboration.

## Discussion

The prevalence of chronic diseases increases with rising life expectancy [33]. Multimorbidity requires coordination between different service providers. Despite its high relevance to care, interactions between T2DM and periodontitis have not yet been comprehensively addressed in current standard care - interdisciplinary care at the interface between medical and dental care has been very limited [34]. There is currently no systematic early detection of periodontitis by general practitioners or of T2DM by dentists using validated screening tools. Standard care lacks a comprehensive therapeutic continuum for integrated disease management of T2DM and periodontitis.

To this end, DigIn2Perio aims to reduce the burden of morbidity and care costs. The implementation of the new care approach will contribute to the quality of care by identifying previously unknown periodontal care needs in patients with T2DM, by reducing the number of undiagnosed patients with T2DM, and by better synchronization of medical and dental care. The new care approach goes well beyond existing standards. It enables systematic screening for T2DM and periodontitis, informs patients about relevant risk factors and useful behavioral changes and initiates risk-oriented integrated care. The possibility of referral from general practitioners to dentists is an innovative expansion of care and is not permitted in current standard care in Germany.

The immediate expected effects of the new implementation approach can be seen in mutual referrals between physicians and dentists, and validated periodontitis and T2DM diagnoses which lead to appropriate therapy. The expected longer-term impacts are improved glycemic control and sustainable periodontal therapy success. In addition, the new form of care leads to an improvement in the quality of life of patients and reduces long-term healthcare costs. Not least, diabetes and periodontitis also depend on socioeconomic factors [35]. Therefore, better integrated care also contributes to equity in care.

Along the lines of learning health systems, the DigIn2Perio study is expected to generate a flow of new evidence that can inform health policy-makers about the potential adoption of the new integrated care model as standard care within the statutory health insurance in Germany, and potentially also in other countries.

## Conclusion

Through evaluating the implementation of a new integrated care concept for diabetes and periodontitis patients, the DigIn2Perio study will generate unique and novel evidence about the extent to which structured screening for T2DM and periodontitis as well as enhanced information exchange between dentists and physicians leads to better alignment of interprofessional care processes, earlier detection and treatment of T2DM and/or periodontitis, and ultimately reduced disease burden and treatment costs. By providing insights into the implementation of integrated T2DM-periodontitis care, the findings of the study are expected to be pivotal for enhancing the integration of oral health in primary care.

## Electronic supplementary material

Below is the link to the electronic supplementary material.

Supplementary Material 1

Supplementary Material 2

Supplementary Material 3

## Data Availability

No datasets were generated or analysed during the current study.

## Abbreviations

BOP: Bleeding on probing
DCE: Discrete Choice Experiment
DigIn2Perio: Digitally Integrated T2DM and Periodontitis
DMP: Disease management program
FAS: Full analysis set
FoPra.HD: Network of research practices at the Heidelberg University Hospital
GP: General Practitioners
HAFO.NRW: Research network of general practitioners in the state of North Rhine-Westphalia
ICER: Incremental cost-effectiveness-relation
ITT: Intention-to-treat
KVBW: Association of Statutory Health Insurance Physicians in the state of Baden-Württemberg
KZVBW: Association of Statutory Health Insurance Dentists in the state of Baden-Württemberg
KZVNR: Association of Statutory Health Insurance Dentists in the state of North Rhine-Westphalia
SD: Standard deviation
SF-36: Short Form 36
T2DM: Type 2 Diabetes Mellitus
