# Supplementary material for: Implementation of integrated care for type 2 diabetes Mellitus and Periodontitis in Germany: study protocol for a practice-based and cluster-randomized trial
Source: BMC Oral Health. 2024 Aug 2;24:879. doi: 10.1186/s12903-024-04672-1 (PMC11297783; doi:10.1186/s12903-024-04672-1)
Supplement: Supplementary file 3 — Supplementary Material 3 [file 12903_2024_4672_MOESM3_ESM.docx]

# Appendix

### Discrete choice experiment

To further support the implementation of the new care approach, a discrete choice experiment (DCE) will identify provider’s payment preferences for integrated diabetes and periodontitis care pathways. An online survey will be carried out to identify whether diabetes and dental care providers prefer certain incentive mechanisms which could contribute to the successful uptake of the intervention in usual care. It will also explore potentially differential preferences between care providers who took part in the clinical trials and those who did not. The number of participants in the DCE is planned to be 300 (150 dentists and 150 GPs; whereof 50% took part in clinical trials, and the other 50% did not). To recruit participants not involved in the clinical trials, an extra sample of 1000 practices will be drawn which are not involved in the clinical trials. Recruitment is planned to take place stratified by dentists, GPs, participation and non-participation in the study. Practices will be contacted in intervals. Contact data of practices not participating in the clinical trials will be identified through manual search in the publicly available provider database of the Association of Statutory Health Insurance Dentists/Physicians. The physicians contacted and recruited for the main study will be informed about the DCE and the separate recruitment before they sign their informed consent form.

### Data protection

#### Access to personal data and pseudonymization/anonymization

Directly person-related data (practices, physicians, assistants, patients) will only be processed between the project partners in the course of the study. Processing will be limited to the smallest possible degree and will only be performed wherever the data can not be processed anonymously or using a pseudonym. This especially applies to names on informed consent forms, direct communication with practices and people working there and to the bank details needed for wire transfer of the incentives and reimbursement. Directly person-related data of participating patients (this especially applies to names on informed consent forms) will be kept in the study folders within each practice and sent to the study director in intervals. Persons not associated with the study will not gain access to directly person-related data, unless required by law.

Data meant to be analyzed will be surveyed pseudonymized using an alphanumeric ID and, as soon as the stage of the study allows it, anonymized. Pseudonymization of practices allows the project partners only to see if the practice is a dental or a general practice, if it is in the intervention or control group and which state the practice is based in. Pseudonymization of patients allows the project partners to assign a patient to practice without knowing who the patient is. Keys to the pseudonyms will be kept at the respective institution of the project partner responsible for the given part of the study (practice pseudonyms) and the respective practices (patient pseudonyms), separate from the gathered data. They will be sent to the study director after the study has ended for archival purposes. Analyzed data will only be published anonymized.

Following §35 2 LDSG BW (data protection regulation in the state of Baden-Württemberg), person-related data will be anonymized as soon as the study allows for it. Regarding the general practitioner and dentist studies, anonymization is planned after the second follow-up T2 ends. Data in the process evaluation will be collected under a pseudonym and will be anonymized after the quantitative survey has ended. Anonymization of the data gathered in the phone interviews will be performed after transcription.

#### Data flow

In a first step, the project partners receive the forms declaring informed consent from the respective practices. Participating practices will further be included in the care contract with TK (described above) via an authorization form that will be sent to the respective project partner and then forwarded to TK.

Practices will recruit patients according to the inclusion criteria and receive their informed consent forms, which then will be forwarded interval-wise to the study administration for archiving. Practices gather data via questionnaires and transfer these to the research database using a patient ID as a pseudonym. The key to decode patient-pseudonyms remains within the respective practice, the project partners will not have access to it. The pseudonymized data in the research database will be checked and forwarded to the project partner responsible for the evaluation.

In the context of the process evaluation, practices will receive written questionnaires. These contain the practice-ID as well as a field for inserting a participant-ID that allows to identify which questionnaires belong to one practice, but not to which person. Data will be collected pseudonymized. Questionnaires will be mailed back to the project partner responsible for the process evaluation. The practice ID will then be enhanced by a random participant ID and analyzed.

#### Storage period

According to the protocol DigIn2Perio ends on 30^th^ September 2026. Data from the general practitioner and dentist study as well as quantitative data from the process evaluation will then be stored and archived for a period of 10 at the study administration. After 10 years, the data will be permanently erased and any sensitive documents will be destroyed. The audio recordings manufactured in the qualitative phase of the process evaluation will be permanently erased after transcription took place. Transcripts will be stored and archived for a period of 10 years at the project partner responsible for the process evaluation. After 10 years, transcripts will be permanently erased.
